# The prevalence and incidence of systemic lupus erythematosus in Taiwan: a nationwide population-based study

**DOI:** 10.1038/s41598-021-84957-5

**Published:** 2021-03-11

**Authors:** Pui-Ying Leong, Jing-Yang Huang, Jeng-Yuan Chiou, Yi-Chiao Bai, James Cheng-Chung Wei

**Affiliations:** 1 Institute of Medicine, Chung Shan Medical University, Taichung, Taiwan, ROC; 2 Division of Allergy, Immunology and Rheumatology, Department of Internal Medicine, Chung Shan Medical University Hospital, Taichung, Taiwan, ROC; 3 Department of Medical Research, Chung Shan Medical University Hospital, Taichung, Taiwan, ROC; 4 School of Health Policy and Management, Chung Shan Medical University, Taichung, Taiwan, ROC; 5 Graduate Institute of Integrated Medicine, China Medical University, Taichung City, Taiwan, ROC

**Keywords:** Immunology, Rheumatology, Risk factors

## Abstract

To estimate the prevalence and incidence rate of systemic lupus erythematosus (SLE) in Taiwan by using a population-based longitudinal database from 2001 to 2011. We conducted a longitudinal Health Insurance Database (LHID) containing 1,000,000 beneficiaries’ records for calculation of prevalence and incidence rate of SLE from 2001–2011. The overall prevalence of SLE in Taiwan in 2011 is 8.11 per 10,000 people with 14.3 per 10,000 people in female and 1.62 per 10,000 people in male. The overall incidence rate of SLE is 0.74–1 per 10,000 person-years with 1.09–1.76 per 10,000 person-years in female and 0.12–0.25 per 10,000 person-years in male. The highest prevalence rate was observed at 40–49 age group in females. There were no significant differences in the overall prevalence among the urban, suburban and rural area in Taiwan while the relative risk is higher in male population living in rural area (RR 1.36, 95% C.I. 1.03–1.79, *p* = 0.0303). The highest income group has a lower relative risk for the prevalence of SLE (RR 0.83, 95% C.I. 0.71–0.97, *p* = 0.0197). The incidence rate of SLE in male in the rural area is also higher than the urban area (RR 2.34, 95% C.I. 1.3–4.22, *p* = 0.0046). Our study covers the longest period among the nation-wide population studies of SLE in Taiwan. The prevalence was increasing especially in the elderly.

## Introduction

Systemic lupus erythematosus (SLE) is a complex autoimmune disease with diverse clinical manifestations involving many organs and systems. It is the third most common autoimmune rheumatic diseases (ARDs) in Taiwan^1^. The overall hazard ratio for mortality rate is 2.20 compared to non-SLE control in previous study by Chen, et al.^2^. The global female to male ratio of SLE ranges from 4.3–13.6, according to different studies^3^. The female: male ratio is 9–12 in Taiwan in previous study by Chiu, et al.^4^. In the United States, the prevalence for SLE was 5.8–130 per 100,000 and the incidence was 1–10 per 100,000 between 1970 and 2000^3,5^. The age- and sex-adjusted incidence of SLE was 2.9 per 100,000 between 1993–2005 in the US^6^. The incidence of SLE was 0.9–3.1 per 100,000 and the prevalence was 4.3–45.3 per 100,000 in Asia–Pacific region^6^. Our study used the Longitudinal Health Insurance Database (LHID) containing 1,000,000 beneficiaries’ records in Taiwanese National Health Insurance Research Database (NHIRD) from 2001–2011 to calculate the sex- and age-specific prevalence and incidence rates of SLE in Taiwan. It is the first study covering the longest period.

## Methods

The National Health Insurance (NHI) service has been launched in Taiwan for over 20 years. It is a single-payer Healthcare Insurance systemic which covers 99% of the Taiwan population. There are approximately 23 million individuals in this system. The NHIRD is a large database maintained by National Health Research Institute. It contains all registry information of the individual beneficiary and LHID is derived from the NHIRD dataset in which 1,000,000 beneficiaries is randomly sampled and is provided for research purposes. All registration and claims data for these 1,000,000 individuals were collected and distributed as LHID 2000. As all personal data in this database had been multiply encrypted, the informed consent was waived by the approving committee. This study complied with relevant laws and regulations, and it was approved by the Chung Shan Medical University Hospital Institutional Review Board (CS15134).

The case ascertainment was based on diagnosis of classification of diseases ninth revision (ICD-9) code 710.0 for SLE in combination with Catastrophic Illness Registry. The approval the Catastrophic Illness certificate of the individual was review by rheumatologists commissioned by the Bureau of National Health Insurance (BNHI). Patients with catastrophic illness certificates are eligible for exemption from co-payments. Therefore, the case ascertainment of SLE patient with catastrophic illness certificate is very reliable.

The economic status was estimated by their insurable monthly incomes, the income was classified into 3 groups (monthly income TWD < 30,000, TWD 30,000–60,000 and TWD ≥ 60,000). The exchange rate is approximately 1:30 (US dollar: Taiwan Dollar). In this study the urbanization was grouped into 3 levels (City, Township, and Rural) like previous study^7^.

The prevalence of SLE was computed for 2001–2011. Incidence was calculated as the number of new cases from 2003 to 2011 divided by the total number of person-years in the available records. The 95% confidence intervals (CI) for incidence were calculated assuming a Poisson distribution. All statistical analyses were performed using SAS statistical software, and the significance level was set at p ≤ 0.05.

## Results

**Figure 1:**
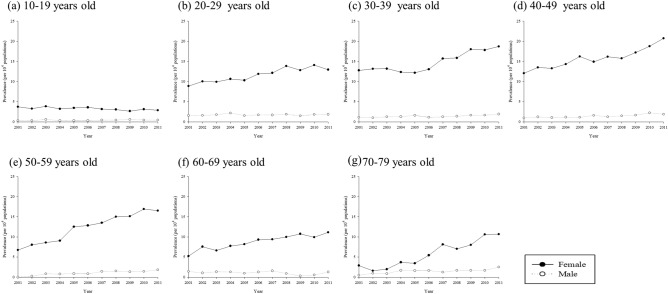
Prevalence of SLE from 2001 to 2011 by age groups.

The prevalence of SLE among 10–79 years old people in Taiwan increased from 2001 to 2011 (Table 1). The overall prevalence ranges from 4.77 to 8.11 per 10,000 people in which female predominant with prevalence about 8.56–14.3 per 10,000 people. The prevalence of SLE in male ranged from 0.91 to 1.62 per 10,000 people. The prevalence trend was steady in the age group of 10–19 (Fig. 1a) in 2001 through 2011. However, there were increases in the prevalence rate of SLE in other age groups especially in females (Fig. 1b–g). The relative risk of prevalent cases of SLE for female was 9.14 (95% C.I. 8.36–9.98, *p* < 0.001) (Table 2). The overall prevalence peaked at 40–49 age group (RR 4.72, 95% C.I. 4.14–5.39, *p* < 0.001). Female patients in 40–49 age group also has the highest prevalence (RR 4.86, 95% C.I. 4.23–5.58, *p* < 0.001) while their male counterparts peaked at age 20–29 (RR 4.45, 95% C.I. 2.98–6.63, *p* < 0.001). There was also another peak of prevalent cases of SLE in male at the very old age group 70–79 (RR 3.63, 95% C.I. 2.25–5.87, *p* < 0.001) (Suppl. Information).

**Table 1:** Prevalence (per 10^4^ people) of SLE among 10–79 years old people in Taiwan from 2001 to 2011.

Year	All			Female			Male		
	Population	Case	Prevalence (95% C.I.)	Population	Case	Prevalence (95% C.I.)	Population	Case	Prevalence (95% C.I.)
2001	778,103	371	4.77 (4.31–5.28)	392,460	336	8.56 (7.69–9.53)	385,643	35	0.91 (0.65–1.26)
2002	789,642	408	5.17 (4.69–5.69)	397,925	371	9.32 (8.42–10.32)	391,717	37	0.94 (0.68–1.30)
2003	814,451	435	5.34 (4.86–5.87)	417,117	391	9.37 (8.49–10.35)	397,334	44	1.11 (0.82–1.49)
2004	821,388	455	5.54 (5.05–6.07)	419,710	407	9.70 (8.8–10.69)	401,678	48	1.19 (0.90–1.59)
2005	864,560	508	5.88 (5.39–6.41)	439,144	460	10.47 (9.56–11.48)	425,416	48	1.13 (0.85–1.50)
2006	860,999	532	6.18 (5.68–6.73)	439,640	483	10.99 (10.05–12.01)	421,359	49	1.16 (0.88–1.54)
2007	859,730	577	6.71 (6.19–7.28)	440,212	526	11.95 (10.97–13.01)	419,518	51	1.22 (0.92–1.60)
2008	859,891	603	7.01 (6.47–7.6)	440,202	548	12.45 (11.45–13.54)	419,689	55	1.31 (1.01–1.71)
2009	851,996	620	7.28 (6.73–7.87)	435,757	567	13.01 (11.98–14.13)	416,239	53	1.27 (0.97–1.67)
2010	861,288	675	7.84 (7.27–8.45)	440,785	614	13.93 (12.87–15.08)	420,503	61	1.45 (1.13–1.86)
2011	862,136	699	8.11 (7.53–8.73)	441,393	631	14.30 (13.22–15.46)	420,743	68	1.62 (1.27–2.05)
p for trend			< 0.0001			< 0.0001			< 0.0001

**Table 2:** Relative risk of prevalent cases of SLE in Taiwan from 2001 to 2011 by using multiple Poisson regression*.

	All		Female		Male	
	RR (95% C.I.)	p value	RR (95% C.I.)	p value	RR (95% C.I.)	p value
Year (per 1 year)	1.05(1.05–1.06)	< .0001	1.05(1.04–1.06)	< .0001	1.05(1.02–1.08)	0.0002
**Sex (ref: Male)**						
Female	9.14(8.36–9.98)	< .0001	–	–	–	–
**Age(ref: 10–19 years old)**						
20–29	3.63(3.17–4.14)	< .0001	3.55(3.08–4.09)	< .0001	4.45(2.98–6.63)	< .0001
30–39	4.45(3.91–5.08)	< .0001	4.56(3.97–5.24)	< .0001	3.44(2.29–5.17)	< .0001
40–49	4.72(4.14–5.39)	< .0001	4.86(4.23–5.58)	< .0001	3.49(2.32–5.24)	< .0001
50–59	3.73(3.25–4.28)	< .0001	3.85(3.33–4.44)	< .0001	2.62(1.69–4.04)	< .0001
60–69	2.66(2.28–3.11)	< .0001	2.67(2.26–3.15)	< .0001	2.60(1.61–4.20)	< .0001
70–79	2.04(1.70–2.46)	< .0001	1.85(1.51–2.26)	< .0001	3.63(2.25–5.87)	< .0001
**Residential urbanization (ref: urban)**						
Sub-urban	0.95(0.90–1.01)	0.1046	0.96(0.90–1.02)	0.1813	0.91(0.75–1.10)	0.3244
Rural	0.99(0.89–1.09)	0.7691	0.95(0.85–1.05)	0.3272	1.36(1.03–1.79)	0.0303
**Income (ref: < 30,000)**						
30,000–60,000	0.96(0.90–1.03)	0.2593	0.96(0.89–1.04)	0.2966	1.03(0.82–1.28)	0.8259
≥ 60,000	0.83(0.71–0.97)	0.0197	0.78(0.65–0.93)	0.0070	1.19(0.86–1.66)	0.2964

*Models were adjusted for calendar year, sex, age groups, urbanization and income levels.

**Figure 2:**
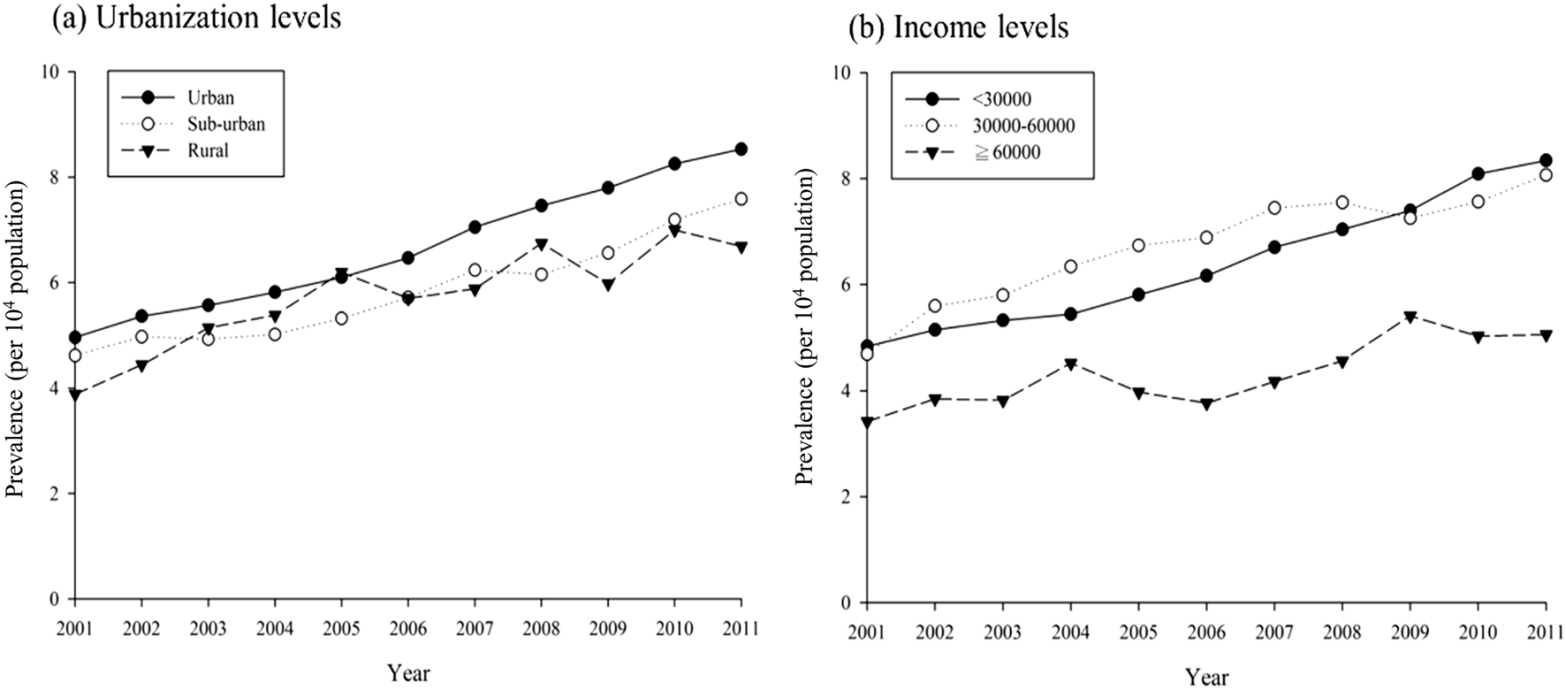
Prevalence of SLE from 2001 to 2011 by **(a)** urbanization levels and **(b)** income levels.

There was no significant difference in patients living in different levels of urbanization except for higher relative risk in male patients living in rural area (RR 1.36, 95% C.I. 1.03–1.79, *p* = 0.0303). For people with highest income group (≥ TWD 60,000/month), the overall relative risk was lower (RR 0.83, 95% C.I. 0.71–0.97 *p* = 0.197). Female with higher income also had relatively lower prevalence of SLE (RR 0.78, 95% C.I. 0.65–0.93, *p* = 0.007). Nevertheless, male in the highest income group had no significance in risk reduction. The prevalence of SLE increased despite urbanization levels from 2001 to 2011 (Fig. 2a) and it also increased in all income levels (Fig. 2b).

In Table 3, the incidence rates of SLE from 2003 to 2011 among 16–79 years old patients were 0.87 per 10,000 person-years (95% C.I. 0.69–1.10), 0.93 per 10,000 person-years (95% C.I. 0.74–1.16), 0.95 per 10,000 person-years (95% C.I. 0.76–1.18), 1(95% C.I. 0.81–1.23), 0.91 per 10,000 person-years (95% C.I. 0.73–1.13), 0.88 per 10,000 person-years (95% C.I. 0.71–1.11), 0.88 per 10,000 person-years (95% C.I. 0.7–1.1), 0.69 per 10,000 person-years (95% C.I. 0.53–0.89) and 0.74 per 10,000 person-years (95% C.I. 0.58–0.95). The incidence rates per 10,000 person-years of SLE for female from 2003 to 2011 were 1.46(95% C.I. 1.14–1.88), 1.60(95% C.I. 1.26–2.03), 1.76(95% C.I. 1.4–2.19), 1.75 (95% C.I. 1.4–2.19), 1.57(95% C.I. 1.24–1.99), 1.55(95% C.I. 1.22–1.96), 1.52(95% C.I. 1.19–1.93), 1.09(95% C.I. 0.82–1.45) and 1.29(95% C.I. 1–1.68). The incidence rates per 10,000 person-years of SLE for male from 2003 to 2011 among 16–79 were 0.25(95% C.I. 014–0.47), 0.22(95% C.I. 0.12–43), 0.12(95% C.I. 0.05–0.28), 0.21(95% C.I. 0.11–0.41), 0.21(95% C.I. 0.11–0.41), 0.19(95% C.I. 0.1–0.38), 0.22(95% C.I. 0.11–0.42), 0.26(95% C.I. 0.14–0.47) and 0.17(95% C.I. 0.08–0.35).

**Table 3:** The incidence rate (per 10^4^ person years) of SLE among 10–79 years old people in Taiwan from 2003 to 2011 by specific sub-groups.

	All			Female			Male		
	Py*	New case	Incidence rate* (95% C.I.)	Py*	New case	Incidence rate* (95% C.I.)	Py*	New Case	Incidence rate* (95% C.I.)
Overall (2001–2011)	7,649,831	667	0.87(0.81–0.94)	3,907,959	590	1.51(1.39–1.64)	3,741,872	77	0.21(0.16–0.26)
**Year**									
2003	813,986	71	0.87(0.69–1.10)	416,695	61	1.46(1.14–1.88)	397,291	10	0.25(0.14–0.47)
2004	820,866	76	0.93(0.74–1.16)	419,240	67	1.60(1.26–2.03)	401,626	9	0.22(0.12–0.43)
2005	863,945	82	0.95(0.76–1.18)	438,591	77	1.76(1.40–2.19)	425,354	5	0.12(0.05–0.28)
2006	860,316	86	1.00(0.81–1.23)	439,020	77	1.75(1.40–2.19)	421,296	9	0.21(0.11–0.41)
2007	858,974	78	0.91(0.73–1.13)	439,524	69	1.57(1.24–1.99)	419,450	9	0.21(0.11–0.41)
2008	859,076	76	0.88(0.71–1.11)	439,459	68	1.55(1.22–1.96)	419,617	8	0.19(0.10–0.38)
2009	851,135	75	0.88(0.70–1.10)	434,972	66	1.52(1.19–1.93)	416,163	9	0.22(0.11–0.42)
2010	860,361	59	0.69(0.53–0.89)	439,940	48	1.09(0.82–1.45)	420,421	11	0.26(0.14–0.47)
2011	861,172	64	0.74(0.58–0.95)	440,518	57	1.29(1.00–1.68)	420,654	7	0.17(0.08–0.35)
p for trend			< 0.001			< 0.001			< 0.001
**Age**									
10–19	1,218,126	76	0.62(0.50–0.78)	590,017	67	1.14(0.89–1.44)	628,109	9	0.14(0.07–0.28)
20–29	1,330,571	131	0.98(0.83–1.17)	734,199	120	1.63(1.37–1.95)	596,372	11	0.18(0.10–0.33)
30–39	1,468,298	144	0.98(0.83–1.15)	746,607	134	1.79(1.52–2.13)	721,691	10	0.14(0.07–0.26)
40–49	1,441,912	128	0.89(0.75–1.06)	720,467	114	1.58(1.32–1.90)	721,445	14	0.19(0.11–0.33)
50–59	1,128,742	95	0.84(0.69–1.03)	570,722	79	1.38(1.11–1.73)	558,020	16	0.29(0.18–0.47)
60–69	624,905	46	0.74(0.55–0.98)	325,050	39	1.20(0.88–1.64)	299,855	7	0.23(0.11–0.49)
70–79	437,277	47	1.07(0.81–1.43)	220,897	37	1.67(1.21–2.31)	216,380	10	0.46(0.25–0.86)
**Urbanization**									
Urban	4,747,328	427	0.90(0.82–0.99)	2,481,290	386	1.56(1.41–1.72)	2,266,038	41	0.18(0.13–0.25)
Sub-urban	2,268,829	176	0.78(0.67–0.90)	1,113,881	156	1.40(1.20–1.64)	1,154,948	20	0.17(0.11–0.27)
Rural	633,674	64	1.01(0.79–1.29)	312,788	48	1.53(1.16–2.04)	320,886	16	0.50(0.31–0.81)
**Income**									
< 30,000	6,071,096	554	0.91(0.84–0.99)	3,255,395	488	1.50(1.37–1.64)	2,815,701	66	0.23(0.18–0.30)
30,000–60,000	1,247,047	91	0.73(0.59–0.90)	559,894	83	1.48(1.20–1.84)	687,153	8	0.12(0.06–0.23)
≥ 60,000	331,688	22	0.66(0.44–1.01)	92,670	19	2.05(1.31–3.21)	239,018	3	0.13(0.04–0.39)

The overall incidence rate for different age groups ranged from 0.62 to 1.07 per 10,000 with highest incidence at age 70–79, while the highest incidence rate for female was at age 30–39 (1.79 per 10,000 person-years, 95% C.I. 1.52–2.13). The highest incidence rate for male was at age 70–79 (0.46 per 10,000 person-years, 95% C.I. 0.25–0.86). For rural area, the incidence rate for SLE was the highest among the three different levels of urbanization (1.01 per 10,000 person-years, 95% C.I. 0.79–1.29). However, for female population, those who live in urban area had higher incidence rate (1.56 per 10,000 person-years 95% C.I. 1.16–2.04) while male has the highest incidence rate in rural area (0.5 per 10,000 person-years 95% C.I. 0.31–0.81). For income difference, lowest earner group has highest overall incidence rate per 10,000 person-years (0.91, 95% C.I. 0.84–0.99). While female with highest income group has a higher incidence rate 2.05 per 10,000 person-years (95% C.I. 1.31–3.21).

**Figure 3:**
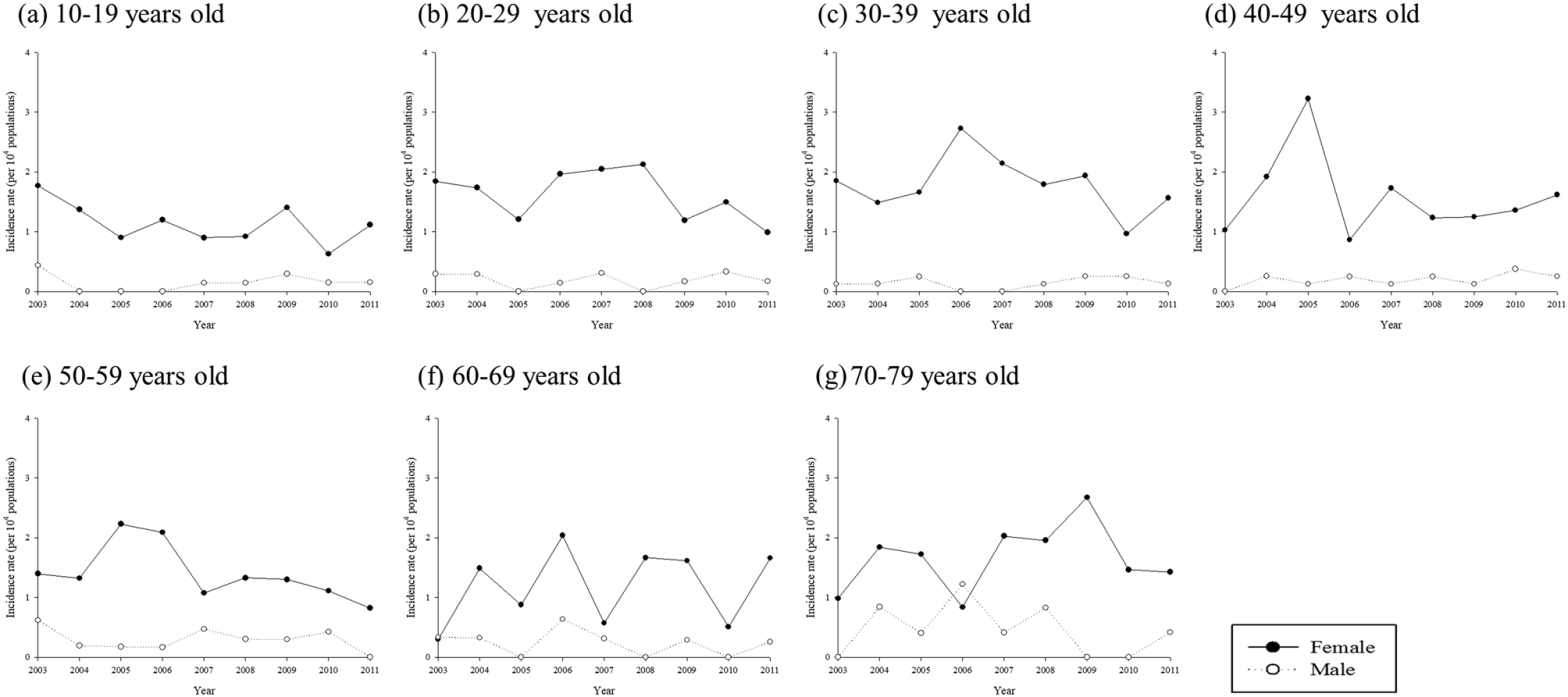
Incidence rate of SLE from 2001 to 2011 by age groups.

Figure 3a–g showed different incident rates in different age groups through 2003–2011. The relative risk of new cases of SLE for female was 7.28(95% C.I 5.73–9.24) (Table 4). The relative risks for overall and female were high in both age 30–39 and 40–49 age groups. However, male patient living in rural area has a higher relative risk of incidence (2.31 95% C.I. 1.3–4.22, *p* = 0.0046). There was no significant difference in relative risk of new cases among people with different groups of income.

**Table 4:** Relative risk of new cases of SLE in Taiwan from 2003 to 2011 by using multiple Poisson regression*.

	All		Female		Male	
	RR (95% C.I.)	p value	RR (95% C.I.)	p value	RR (95% C.I.)	p value
Year (per 1 year)	0.97(0.94–1.00)	0.0638	0.97(0.94–1.00)	0.0488	1.00(0.91–1.09)	0.9394
**Sex (ref: male)**						
Female	7.28(5.73–9.24)	< .0001	–	–	–	–
**Age (ref: 10–19 years old)**						
20–29	1.45(1.09–1.93)	0.0098	1.44(1.07–1.95)	0.0168	1.44(0.59–3.48)	0.4218
30–39	1.57(1.18–2.08)	0.0020	1.59(1.18–2.15)	0.0024	1.21(0.48–3.01)	0.6901
40–49	1.42(1.06–1.89)	0.0178	1.39(1.02–1.88)	0.0372	1.65(0.70–3.87)	0.2501
50–59	1.35(0.99–1.83)	0.0567	1.23(0.89–1.71)	0.2139	2.41(1.05–5.54)	0.0377
60–69	1.13(0.78–1.63)	0.5212	1.07(0.72–1.58)	0.7487	1.65(0.61–4.44)	0.3232
70–79	1.66(1.15–2.40)	0.0063	1.49(1.00–2.23)	0.0515	2.98(1.21–7.36)	0.0179
**Residential urbanization (ref: urban)**						
Sub-urban	0.90(0.76–1.08)	0.2644	0.91(0.75–1.09)	0.3128	0.92(0.54–1.57)	0.7518
Rural	1.17(0.89–1.52)	0.2535	1.01(0.74–1.36)	0.9740	2.34(1.30–4.22)	0.0046
**Income (ref: < 30,000)**						
30,000–60,000	0.87(0.69–1.09)	0.2286	0.93(0.73–1.18)	0.5289	0.54(0.25–1.15)	0.1085
≥ 60,000	1.11(0.71–1.71)	0.6531	1.29(0.81–2.06)	0.2799	0.54(0.16–1.75)	0.3006

*Models were adjusted for calendar year, sex, age groups, urbanization and income levels.

## Discussion

Our study showed that the incidence of SLE is 0.81–1 per 100,000 person-year and the prevalence is 8.11 per 100,000 between 2001 and 2011. In this study, the female to male ratio of prevalence of SLE is 9.14 per 100,000 and the female to male rate in incidence rate is 7.28 per 100,000. That is similar to previous studies (4.3–13.6 per 100,000) by Petri, et al. ^3^ and also similar to previous study 8.5 by See, et al.^1^. For people in the highest income group, TWD ≥ 60,000/month, the prevalence is lower in overall and in female patients but not in male patients. This might be due to relatively low number of male patients in SLE. The relation of prevalence, the location and socioeconomic in SLE patients especially in male were not mentioned in other studies in Taiwan. Although the incidence rate varied in different years, we found that the incidence rate was not increasing in Taiwan unlike many other countries^8–12^. Chiu, et al. found that the incidence decreased steadily from 0.99 per 10,000 per year in 2001 to 0.68 per 10,000 per year in 2007^4^. In our study, the incidence rate in 2007 were 0.91 per 10,000 per year, which is very similar to Chiu’s study. In our study, the trend increased at first from 2003 to 2007 but there is no significance for p value with 95% CI. We also see the decrease in trend in 2010 and 2011 which is significance with the 95% CI. However, owing to low incidence rate, it was difficult to conclude that the incidence increased in 2003 to 2007 and then decreased in 2010 to 2011. Further studies should be done but our dataset only included the patients from 2000 to 2011. SLE affects mostly premenopausal women. However, we also found that there was another peak of incidence rate in the very old age group (age 70–79). This was not reported in previous studies and this result might be due to longer observation period in our studies, increased awareness of SLE, the development of standardized classification criteria and easier access to rheumatologist care^13^.

The earliest study of the epidemiology of rheumatic disease (RD) in Taiwan was by Chou, et al. in 1994^14^. Although it analyzed different rheumatic diseases (RDs) such as rheumatoid arthritis (RA), osteoarthritis (OA), ankylosing spondylitis (AS) and gout by regions, it did not include SLE patients as the prevalence rate was low and the study population was relatively small. That was before 2015 when the National Health Insurance was launched in Taiwan^15^. Then Chiu, et al. used the NHIRD dataset from 2000 to 2007 for epidemiology study specifically for SLE^4^. In 2013, Yeh, et al. tried to find out the burden of SLE in Taiwan using the NHIRD from 2003 to 2008 and added the mortality rate but without further analysis of the urbanization and economic status of the patients^16^. See and Kuo, et al. used the same dataset to see the epidemiology of RDs 2005–2009 and 2000–2008 where SLE is the third and the second highest incidence rate among the rheumatic diseases in Taiwan^1,17^. Our study specifically looked at the prevalence and incidence of SLE in Taiwan and analyzed the SLE patients with different socioeconomic status and locations. Our studies covered the longest period and provided more details about the impact of locations and incomes on the incidence of SLE.

Socioeconomic status is associated with higher mortality rate and poorer prognosis of SLE. However, previous study in Sweden showed that unemployment, dismissal, and severe economic problems dose not associated with the incidence of SLE^18^. Another study by Koreans, also showed that the income levels did not affect the incidence or prevalence of SLE^19^. In our study, the prevalence of SLE was lower in high income level but was only significant in female in Table 2 (*p* = 0.0070). The finding was a little bit different from those in the Swedish and Korean groups. The Canadian group by George, et al., studied the socioeconomic status of patients with SLE by the surrogate of education levels as they thought the damage from SLE might affect the working ability and the income of the patients^20^. In our study, the education levels of the patients were not recorded. We used the method from previous Taiwan NHRD studies by estimating the income levels of SLE patients. The Taiwan National Health Insurance premiums for individuals are calculated based on the monthly income they report to the National Health Insurance Administration. Therefore, in our study, the economic status of the patients was estimated by premium insured and classified into three groups (< 30,000, 30,000–60,000 and ≥ 60,000 New Taiwan Dollars [NTD]) by the exchange rate (about 1 USD = 30 NTD). The premiums of the low-income household members are 100% supported by the Government. We calculate the prevalent cases and incidence of SLE so that only newly diagnosed SLE was counted in each year. Therefore, the damage of SLE which might influence the patients’ ability of work was minimized. Another study about the socioeconomic status of rheumatoid arthritis patients published in 2018 also used this definition for economic levels^21^. Our study suggested that the prevalence and incidence for SLE is lower in higher income levels group. In Table 3, the lower income earners had the highest overall incidence rate of SLE. However, in Table 4, when the 2nd and the highest earner compared with the reference (< 30,000 NTD group), there was no significant difference. It might be due to small size in the number of patients with SLE in these two groups.

The definition of urbanization levels was modified from the method by Liu, et al.^22^. The level of urbanization was originally divided into seven categories according to demographic components in communities. In this study, urbanization was regrouped into three levels (urban, suburban, and rural areas), as in previous studies^13,21^.

It is our limitation that the location of the beneficiaries was classified according to the insured locations as a proxy for distinguishing the urban, suburb and rural area. According to the Taiwan Health Insurance Act, Chapter 2, Article 10, the insured shall be classified into six categories ^23^. The Categories 1 to 4 are people who have jobs. In Article 15, “ the group insurance applicants Categories 1 and 2, the group insurance applicants shall be the agencies, schools, enterprises, institutions, or employers, which they work for, or unions where they hold membership. For the insured in Category 3, the group insurance applicants shall be the lowest-level Farmers Association, Irrigation Association or Fishers Association to which they belong, or located at the place where the insured have their household registered. For the insured in Categories 5 and 6, the group insurance applicants shall be the village (township, municipal, district) administration offices of their registered domiciles; provided, however, the public or private social welfare service institutions may be the group insurance applicants for the insured who lives therein”. Therefore, we assumed that most of the beneficiaries lived in or near the location of insured. Further validation of the NIH studies would be needed to prove our assumption and we know that there may be a selection bias for categorizing urban, suburb or rural areas. We could not obtain the information of the duration of the beneficiaries living in the location so we could not analyze impact of duration of living in a certain place on the prevalence and incidence of the disease. Notably, the increase in prevalence and incidence rate in the male living in the rural area in our study might be speculated as male living in rural areas usually worked in the primary industries, e.g. farming and fishing. The increase risk of SLE may be due to the exposure of sunlight which is the environmental trigger of SLE^24,25^. Further study is needed to exemplify this.

The strength of our study is a very long observational period and the database set is nation-wide and could almost fully represent Taiwan as the NHIRD covers nearly 99% of the population. The case ascertainment is very accurate by using the ICD-9 code of 710.0 together with Catastrophic Illness Registry as it was issued after reviewing by peer rheumatologist. In the age group analysis, we found that the incidence is increasing in the elderly group and those who live in the rural area has a higher relative risk for male. Unlike other study by Chakravarty, et al. that the urban region has a high incidence rate of SLE ^26^. These two findings were not observed before and the reason could not be fully explained. It might be due to the availability of medical resources were relatively accessible even though in rural area in Taiwan.

The limitation of our study is that we could not access the severity and activities of the disease as these were not included in the dataset. As for ethnicity, this dataset is from Taiwan’s NHIRD study and comprised of the Taiwanese population, so the lack of ethnic information is indeed another limitation in this study. We did not analyze the survival rate, the comorbidity and mortality of the disease.

## Conclusion

Our results are compatible with previous findings on the sex ratio distribution. This is the first study to about the trend of SLE in Taiwan and it is the first study investigates into the prevalence and incidence rate of SLE in different age groups, income levels and residential areas. The incidence in SLE is stable and it occurs predominantly in 20–49 age groups.

## Supplementary Information

Supplementary Figures.

## References

[1] See LC, Kuo CF, Chou IJ (2013). Sex- and age-specific incidence of autoimmune rheumatic diseases in the Chinese population: A Taiwan population-based study. Semin. Arthritis Rheum..

[2] Chen YM, Lin CH, Chen HH (2014). Onset age affects mortality and renal outcome of female systemic lupus erythematosus patients: A nationwide population-based study in Taiwan. Rheumatology (Oxford).

[3] Petri M (2002). Epidemiology of systemic lupus erythematosus. Best. Pract. Res. Clin. Rheumatol..

[4] Chiu YM, Lai CH (2010). Nationwide population-based epidemiologic study of systemic lupus erythematosus in Taiwan. Lupus.

[5] Uramoto KM, Michet CJ, Thumboo J (1999). Trends in the incidence and mortality of systemic lupus erythematosus, 1950–1992. Arthritis Rheum..

[6] Borchers AT, Naguwa SM, Shoenfeld Y (2010). The geoepidemiology of systemic lupus erythematosus. Autoimmun. Rev..

[7] Alamanos Y, Voulgari PV, Siozos C (2003). Epidemiology of systemic lupus erythematosus in northwest Greece 1982–2001. J. Rheumatol..

[8] Rees F, Doherty M, Grainge M (2016). The incidence and prevalence of systemic lupus erythematosus in the UK, 1999–2012. Ann. Rheum. Dis..

[9] Carter EE, Barr SG, Clarke AE (2016). The global burden of SLE: Prevalence, health disparities and socioeconomic impact. Nat. Rev. Rheumatol..

[10] Walsh SJ, Gilchrist A (2006). Geographical clustering of mortality from systemic lupus erythematosus in the United States: Contributions of poverty, hispanic ethnicity and solar radiation. Lupus.

[11] Pons-Estel GJ, Alarcon GS, Scofield L (2010). Understanding the epidemiology and progression of systemic lupus erythematosus. Semin. Arthritis Rheum..

[12] Jarukitsopa S, Hoganson DD, Crowson CS (2015). Epidemiology of systemic lupus erythematosus and cutaneous lupus erythematosus in a predominantly white population in the United States. Arthritis Care Res. (Hoboken).

[13] Hsu CC, Lee CH, Wahlqvist ML (2012). Poverty increases type 2 diabetes incidence and inequality of care despite universal health coverage. Diabetes Care.

[14] Chou CT, Pei L, Chang DM (1994). Prevalence of rheumatic diseases in Taiwan: A population study of urban, suburban, rural differences. J. Rheumatol..

[15] Hsieh CY, Su CC, Shao SC (2019). Taiwan's National Health Insurance Research Database: Past and future. Clin. Epidemiol..

[16] See LC, Kuo CF, Chou IJ (2013). Sex- and age-specific incidence of autoimmune rheumatic diseases in the Chinese population: A Taiwan population-based study. Semin. Arthritis Rheum..

[17] Yeh K, Yu C, Chan P (2013). Burden of systemic lupus erythematosus in Taiwan: A population-based survey. Rheumatol. Int..

[18] Bengtsson AA, Rylander L, Hagmar L (2002). Risk factors for developing systemic lupus erythematosus: A case–control study in southern Sweden. Rheumatology.

[19] Bae EH, Lim SY, Han KD (2020). Trend of prevalence and incidence of systemic lupus erythematosus in South Korea, 2005 to 2015: A nationwide population-based study. Korean J. Intern. Med..

[20] George A, Wong-Pak A, Peschken CA (2017). Influence of education on disease activity and damage in systemic lupus erythematosus: Data from the 1000 canadian faces of lupus. Arthritis Care Res..

[21] Yang DH, Huang JY, Chiou JY (2018). Analysis of socioeconomic status in the patients with rheumatoid arthritis. Int. J. Environ. Res. Public Health..

[22] Liu CY, Hung YT, Chuang Y (2006). Incorporating development stratification of Taiwan townships into sampling design of large scale health interview survey. J. Health Manag..

[23] *Law and Regulations Database of the Republic of China, National Health Insurance Act*, announced date: 1994-08-09.

[24] Barbhaiya M, Costenbader KH (2014). Ultraviolet radiation and systemic lupus erythematosus. Lupus.

[25] Wolf SJ, Estadt SN, Gudjonsson JE (2018). Human and murine evidence for mechanisms driving autoimmune photosensitivity. Front. Immunol..

[26] Chakravarty EF, Bush TM, Manzi S (2007). Prevalence of adult systemic lupus erythematosus in California and Pennsylvania in 2000: Estimates obtained using hospitalization data. Arthritis Rheum..

