# Supplementary material for: The prevalence and incidence of systemic lupus erythematosus in Taiwan: a nationwide population-based study
Source: Sci Rep. 2021 Mar 11;11:5631. doi: 10.1038/s41598-021-84957-5 (PMC7952694; doi:10.1038/s41598-021-84957-5)
Supplement: Supplementary file 1 — Supplementary Figures. [file 41598_2021_84957_MOESM1_ESM.docx]

**Title**

The Prevalence and Incidence of Systemic Lupus Erythematosus in Taiwan: A Nationwide Population-based Study

**Author List**

Pui-Ying Leong^1,2^, Jing-Yang Huang^1,3^, Jeng-Yuan Chiou^1,4^, Yi-Chiao Bai^1^, James Cheng-Chung Wei^1,2,5^*

^1^ Institute of Medicine, Chung Shan Medical University, Taichung, Taiwan, Republic of China

^2^Division of Allergy, Immunology and Rheumatology, Department of Internal Medicine, Chung Shan Medical University Hospital, Taichung, Taiwan, Republic of China

^3^Department of Medical Research, Chung Shan Medical University Hospital, Taichung, Taiwan, Republic of China

^4^School of Health Policy and Management, Chung Shan Medical University, Taichung, Taiwan, Republic of China

5 Graduate Institute of Integrated Medicine, China Medical University, Taichung, Taiwan.

*Correspondence: James Cheng-Chung Wei, MD, PhD.

Division of Allergy, Immunology and Rheumatology, Chung Shan Medical University Hospital; Institute of Medicine, Chung Shan Medical University; Graduate Institute of Integrated Medicine, China Medical University, Taichung, Taiwan.

No. 110, Sec. 1, Jianguo N. Rd., South District, Taichung City 40201, Taiwan. (TEL)＋886 4 24739595 #34718. E-mail: [jccwei@gmail.com](mailto:jccwei@gmail.com)





Supplementary Figure 1. Prevalence of SLE in different age group.





Supplementary Figure 2. Incidence of SLE in different age group.
